# Adult female callers’ characteristics and mental health status: a retrospective study based on the psychological assistance hotline in Hangzhou

**DOI:** 10.1186/s12889-023-17085-6

**Published:** 2023-11-20

**Authors:** Yating Wei, Haidong Song

**Affiliations:** 1 School of Mental Health and Psychological Sciences, Anhui Medical University, Hefei, 230032 China; 2 Present Address: Affiliated Mental Health Center & Hangzhou Seventh People’s Hospital, Zhejiang University School of Medicine, Hangzhou, 310013 China

**Keywords:** Psychological assistance hotline, Female callers, Mental health status

## Abstract

**Objective:**

This study aims to analyze the basic characteristics and mental health status of adult female callers to the psychological assistance helpline in Hangzhou City, in order to provide targeted services for effectively intervening in the psychological crises of this group.

**Methods:**

Data from adult female callers to a helpline in Hangzhou City were collected between 2019 and 2022, encompassing demographic information and discussed issues. The data were analyzed according to age groups, marital status, and call times. The mental health status of the adult female population was measured by two indicators—mood status and suicide risk.

**Results:**

The study included 15,580 adult female callers. Among them, 52.2% were aged 30 and below, 62.1% were unmarried, and 42.6% were from other provinces. The primary types of calls were related to mental health (56.5%), romantic relationships (11%), and marriage/family issues (13%). Adults aged 30 and below had more consultations about romantic relationships, work, and study-related issues compared to those over 30. Those over 30 sought more advice on marriage/family and child education. Younger callers displayed higher levels of depression and suicide risk compared to older callers. Unmarried callers had a higher proportion of moderate depression and suicide risk than married callers. Higher education levels were associated with lower depression levels (OR = 0.631,95%CI:0.439–0.906, *P* = 0.013) and high-risk proportions (OR = 0.328,95%CI:0.147–0.733, *P* = 0.007). Late evening callers had a higher high-risk proportion (OR = 5.326,95%CI:2.633–10.775, *P* < 0.001), and employed individuals had lower high-risk proportions compared to unemployed callers (OR = 0.536,95%CI:0.320–0.897, *P* = 0.018).

**Conclusion:**

The mental health status of female callers aged 30 and below, unemployed individuals, and those calling in the latter part of the night have relatively poorer mental health and are more likely to be at risk for suicide, which needs to be taken seriously, and more professional and targeted intervention services need to be enhanced in the hotline.

**Supplementary Information:**

The online version contains supplementary material available at 10.1186/s12889-023-17085-6.

According to data from the World Health Organization (WHO) [1], depression is the second largest global epidemic after cardiovascular disease, affecting over 300 million people. Since 2010, in China, depression has been the second leading cause of years lived with disability. The latest Chinese mental health survey results indicate a lifetime prevalence of 6.8% for adult depression disorders in China [2]. The prevalence rates of major depressive disorder, dysthymia, and unspecified depressive disorder are 3.4%, 1.4%, and 3.2%, respectively. When considering any subtype of depression, the weighted lifetime prevalence is 8.0% for females and 5.7% for males, indicating that females are 1.44 times more likely to be affected. Additionally, women exhibit higher rates of suicide and suicidal behaviors compared to men [3].

Research suggests that women are more prone to experiencing negative emotions in response to stressful events [4], particularly under the combined influence of physiological and cognitive factors. Taking the recent COVID-19 pandemic as an example, multiple studies have demonstrated that women have been disproportionately affected during the pandemic [5]. In 2020, there were a total of 374 million cases of anxiety disorders worldwide, with nearly 76 million new cases attributed to the COVID-19 pandemic. Among these new cases, women accounted for nearly 52 million, while men accounted for only 24 million [6]. Another study presented in the Headway 2023 Mental Health Index showed that 83% of women reported mental health issues during the pandemic [7]. Research has shown that the trajectory of change in mental health levels over the past three decades has been different for men and women, with women's mental health improving significantly less overall than men’s, possibly due to more unfavorable physiological and socio-environmental factors slowing down the process of improvement in women’s mental health [8]. According to a research study, the total score of women’s mental health status and the number of positive items differed significantly between genders, and women’s scores on factors such as somatization, depression, anxiety, and fear were higher than men’s, which suggests that they are more fragile and susceptible to the effects of somatization [9].

Gender differences in roles, responsibilities, and workloads within society contribute to variations in the factors that influence anxiety and depression. Specifically, women often face greater conflicts between their family and career responsibilities, which can make them more vulnerable to stress. The traditional patriarchy and the institution of marriage establish a hierarchical gender division of labor, where men hold dominant positions and women are subordinate [10]. This gender division of labor, along with the associated value systems, supports an imbalanced and antagonistic relationship structure.The findings of the Global Gender Gap Report (2013) also indicate that prevailing norms and cultural practices are barriers to women’s access to top leadership/management positions in different countries [11]. Additionally, factors such as the perinatal period [12] and perimenopause [13] can have a significant impact on women's mental health, leading to higher levels of anxiety and depression. Hotlines for mental health support have become an important resource for seeking psychological assistance due to their privacy, speed, accessibility, and convenience. They also serve as a crucial means for individuals at risk of suicide to seek psychological relief and crisis intervention [14]. Previous studies have found that female callers, young people, and unmarried individuals are more prevalent among hotline users [15]. Callers generally have poor mental health, high levels of depression [16], and an increased risk of suicide [17]. However, previous studies tend to use samples from specific groups, such as college students, so it is difficult to generalize to adult women of different ages and education levels. This limits the full understanding of mental health issues.In addition, Previous studies also had problems such as small sample size and insufficient information collection. This study is based on the data form Hangzhou psychological assistance hotline, which provides 24-h free services nationwide, offers psychological support to tens of thousands of callers each year, with half of them being female. That means this study covers a large number of callers, and collects a wealth of caller information, which makes the study with higher statistical reliability and can provide more representative results, and thus has stronger practical guiding value for psychological hotline service and policy formulation.

## Objectives and methods

### Object

This study primarily includes adult female individuals who called a hotline for psychological consultation regarding mental health issues between 2019 and 2022.The inclusion and exclusion criteria are as follows: (1) Adult women over the age of 18; (2) Calls that are invalid, that last less than 600 s, and that are for consultation only are excluded; (3) Exclude repeat calls. A total of 15,580 call records were included in this study. The participants were divided into two age groups based on the age of 30: “aged 30 and below” and “aged above 30. This age division is mainly based on traditional Chinese values that set the roles and life stages of women. Chinese society belongs to the “universal marriage” society, 94% of men and 99% of women get married before the age of 50, 20 to 30 years old is the main age for Chinese people to form a marriage relationship [18]. After 30 years of age are not married women, will face huge pressure to urge marriage from their native families. Besides, It is widely believed that there is an age limit to a woman’s fertility. Heckhausen proposed the concept of development deadlines in life control theory [19]. According to this theory, the development deadline is an age-based limit for achieving a goal [20]. The limitation of a woman’s reproductive age is a kind of biological deadline. Study has shown that women over 30 have lower fertility intention and post-birth recovery ability than women under 30 [21]. Marriage and childbearing, mean that women will enter into a new stage in life, that, for Chinese women is of great significance, especially in their 30 s [22].

The population was further categorized into three education levels: “primary school or below” for individuals with limited education, “high school or vocational school” for individuals with moderate education, and “college or above” for individuals with higher education. These categorizations facilitate data analysis in the later stages of the study.

### Methods

Basic demographic information (gender, age, educational attainment, marital status, occupation, etc.) was collected as general population information. Additionally, during the counseling process, the emotional state, suicide risk, and related factors of the callers were also assessed. As the amount of missing data is minimal and deletion does not result in a significant reduction in the sample size. Therefore, we do the deletion process. The missing values for each variable are as follows: Region = 1; Age = 1; Marital status = 625(4% of the total sample size); Education level = 3; Occupation = 6; Suicide behavior = 1; Suicide plan = 1.

#### Assessment

The evaluation of depressive mood was mainly carried out through a self-made assessment tool suitable for depression, which was developed according to the diagnosis of “major depressive episode” in the fourth edition of the Diagnostic and Statistical Manual of Mental Disorders. The tool has good validity and reliability, and has been widely used in hotline work [23]. Hotline counseling will use the tool to learn about the caller’s depressive symptoms and duration, and the system will automatically calculate a score to assess the caller’s depressive mood over a 2-week period. The caller’s history of suicide attempts is determined by asking if he or she has committed suicide or intentionally harmed himself or herself at any time in the past. If the caller has tried to end his or her life by harming himself or herself in the past, he or she is judged to have a history of suicide attempts.

#### Categorization of consultation issues

Based on the topics raised by callers during hotline consultations, the problems presented by callers can be classified into the following categories: 1) Physical illness, 2) Mental health issues, 3) Romantic relationship issues, 4) Marriage and family issues, 5) Sexual issues, 6) Parenting and education issues, 7) Work-related issues, 8) Interpersonal relationship issues, 9) Learning issues, and 10) Other issues.

#### High-risk call evaluation criteria (Refer to Additional file 1)

Definition of high-risk calls [24, 25]:

- ① Individuals with suicidal thoughts and specific suicide plans.
- ② Individuals with suicidal thoughts but no plan, with a risk factor score ≥ 5.

In addition, the emotional state of the callers is also considered as a risk factor in the scoring system (*Moderate depression is assigned 1 point, and severe depression is assigned 2 points*).

### Statistical methods

Statistical analysis was performed using SPSS 26.0 software. Chi-square tests were used to compare the differences in the composition of age and marital status. Two-sample comparisons were conducted for the mental health of callers at different time points, with a significance level set at bilateral* P* < 0.05. By calculating the median score of the “emotional state” variable, the variable is transformed into a binary variable (lighter level = 0; heavier level = 1) as the dependent variable. Demographic data (age, marital status, years of education), employment status, and time of call are included as independent variables in multiple logistic regression to analyze the factors influencing depressive symptoms among female callers. High-risk calls are used as the dependent variable, with demographic data, employment status, and time of call as independent variables in multiple logistic regression to explore the factors affecting the mental health status of this group. A significance level of *P* < 0.05 indicates statistically significant differences.

## Results

### General characteristics of adult female callers

Among 15,580 adult female callers, the overall age distribution is relatively young, with 52.2% of callers aged 30 and below, 47.8% of callers aged above 30. 62.1% of callers are unmarried, while married and divorced/widowed callers account for 23% and 14.9%, respectively. 71.3% of callers have received higher education. In terms of call time, daytime (7-18 h), first half of the night (19-23 h), and second night time (0-6 h) account for 40.6%, 37.1%, and 22.3% of the total calls, respectively. Calls from other provinces accounting for 42.6%, local calls accounting for 34.9%, and calls from the Zhejiang province accounting for 22.4%. The main reasons for calls include mental health issues (56.5%), marital and family issues (13%), and relationship issues (11%) (Refer to Tables 1 and 2).

**Table 1:** Comparison of basic characteristics of female callers based on age group [PCS (%)]

**Variable**	**Total (*n* = 15579)**	**≤ 30**^a^ **(*n* = 8138)**	**> 30 (*n* = 7441)**	${{\varvec{\chi}}}^{2}$	**P**
**Call time**					
Daytime	6327 (40.6%)	2914 (35.8%)	3413 (45.9%)	338.966	< *0.001*
First half of the night	5785 (37.1%)	2958 (36.3%)	2827 (38%)		
Second half of the night	3467 (22.3%)	2266 (27.8%)	1201 (16.1%)		
^a^**Education level**					
Low	1074 (8.2%)	308 (4.4%)	766 (12.6%)	1006.666	< *0.001*
Medium	2683 (20.5%)	882 (12.6%)	1801 (29.5%)		
High	9327 (71.3%)	5799 (83%)	3528 (57.9%)		
^a^**Occupation**					
Unemployed	7270 (51.1%)	4084 (54.6%)	3186 (47.3%)	74.715	< *0.001*
Employed	6946 (48.9%)	3399 (45.4%)	3547 (52.7%)		
^a^**Marital status**					
Married	3436 (23%)	717 (9.2%)	2719 (38%)	5294.637	< *0.001*
Single	9292 (62.1%)	6973 (89.4%)	2319 (32.4%)		
Divorced or widowed	2226 (14.9%)	108 (1.4%)	2118 (29.6%)		
^a^**Region**					
Hangzhou	5383 (34.9%)	1963 (24.5%)	3420 (46.2%)	904.028	< *0.001*
Zhejiang province	3457 (22.4%)	1839 (23%)	1618 (21.9%)		
Other province	6569 (42.6%)	4207 (52.5%)	2362 (31.9%)		

Some variables involved in the study may have missing data, and been marked “^a^”, resulting in varying numbers of callers for each variable, not encompassing the entire number of callers

**Table 2:** Comparison of types of calls from female callers based on age group [PCS (%)]

**Consultation issues**	**Total (*n* = 15579)**	**≤ 30**^a^ **(*n* = 8138)**	**> 30 (*n* = 7441)**	${{\varvec{\chi}}}^{2}$	**P**
Physical illness	143 (0.9%)	66(0.8%)	77(1%)	2.121	*0.145*
Mental health issues	8794 (56.5%)	4551(55.9%)	4243(57%)	0.832	*0.362*
Romantic relationship issues	1707 (11%)	1214(14.9%)	493(6.6%)	243.925	< *0.001*
Marital and family issues	2032 (13%)	877(10.8%)	1155(15.5%)	67.11	< *0.001*
Sexual issues	32 (0.2%)	12(0.1%)	20 (0.3%)	2.785	*0.095*
Parenting and child education issues	361 (2.3%)	10(0.1%)	351 (4.7%)	354.052	< *0.001*
Work-related issues	594 (3.8%)	365(4.5%)	229 (3.1%)	20.198	< *0.001*
Interpersonal relationship issues	754 (4.8%)	408(5%)	346 (4.7%)	1.062	*0.303*
Learning issues	252 (1.6%)	237(2.9%)	15 (0.2%)	176.565	< *0.001*
Other issues	904(5.8%)	395(4.9%)	509 (6.8%)	26.439	< *0.001*

Some variables involved in the study may have missing data, and been marked “^a^”, resulting in varying numbers of callers for each variable, not encompassing the entire number of callers

### Comparison of basic characteristics of female callers of different age groups on the hotline

There are significant differences among callers of different age groups in terms of calling times, education, employment, regional distribution, and marital status. Callers aged 30 and below were more likely to call during late night hours (27.8% vs 16.1%), have higher education (83% vs 57.9%), be unmarried (89.4% vs 32.4%), and come from outside the province (52.5% vs 31.9%). Callers above 30 had a higher employment ratio (52.7% vs 45.4%) and a higher proportion of calls from the local city (46.2% vs 24.5%) (Table 1).

### Comparison of call types by age groups of female callers

There are significant variations in the main issues discussed by female callers of different age groups on the hotline. Callers aged 30 and below seek guidance on Romantic relationship, work, and study-related matters more frequently, while callers above 30 seek advice on marriage, family, and child education at a higher rate. These differences are statistically significant (*P* < 0.001) (Table 2).

### Comparison of suicide risk and mental health status among female callers of different age groups

Significant differences exist in suicide risk and mental health status among female callers of different age groups. Young female callers (aged 30 and below) show higher proportions of depressive emotions and suicidal thoughts, plans, and behavior compared to female callers above 30 (Table 3).

**Table 3:** Comparison of suicide risk and mental health status of female callers based on age group [PCS (%)]

**Mental health factor**	**Total (*n* = 15579)**	**≤ 30**^a^ **(*n* = 8138)**	**> 30 (*n* = 7441)**	${{\varvec{\chi}}}^{2}$	**P**
**Mood status**					
Normal	14927 (95.8%)	7645 (93.9%)	7282 (97.9%)	6.238	*0.013*
Mild depression	185 (1.2%)	130 (1.6%)	55 (0.7%)	24.113	< *0.001*
Moderate depression	202 (1.3%)	165 (2%)	37 (0.5%)	70.2	< *0.001*
Severe depression	265 (1.7%)	198 (2.4%)	67 (0.9%)	53.675	< *0.001*
**Suicide risk**					
Have suicidal ideation	1937 (12.4%)	1464 (18%)	473 (6.4%)	423.061	< *0.001*
Have suicide plan	91 (0.6%)	77 (0.9%)	14 (0.2%)	38.237	< *0.001*
^a^**Suicide behavior**					
No	15056 (96.7%)	7708 (94.7%)	7348 (98.8%)	6.545	*0*
Used to be	474 (3%)	387 (4.8%)	87 (1.2%)	164.307	< *0.001*
Preparing	12 (0.1%)	12 (0.1%)	0 (0%)	10.972	*0.001*
Already	35 (0.2%)	31 (0.4%)	4 (0.1%)	18.52	< *0.001*
**High-risk call**	121 (0.8%)	101 (1.2%)	20 (0.3%)	47.312	< *0.001*

Some variables involved in the study may have missing data, and been marked “^a^”, resulting in varying numbers of callers for each variable, not encompassing the entire number of callers

### Comparison of basic characteristics of adult female callers by marital status

Marital status significantly affects the characteristics of adult female callers. Married and unmarried women tend to call at night, while divorced/widowed women call during the daytime. Divorced/widowed women have lower education levels. The proportion of unmarried and married women with higher education is 81% and 61.9%, respectively. Married women have a higher proportion of low education (16.3%) compared to divorced/widowed women (12.4%) and unmarried women (4.7%). The employment rate is highest among unmarried women. However, married women have a significantly higher employment rate at 67.3% compared to unmarried women (46.2%) and divorced/widowed women (33.3%). In terms of callers from different region. The proportion of married, unmarried, divorced or widowed women was statistically different (Table 4).

**Table 4:** Comparison of basic characteristics of adult female callers on marital status [PCS (%)]

**Variable**	**Total (*n* = 14955)**	^a^**Married (*n* = 3436)**	^a^**Single (*n* = 9293)**	^a^**Divorced or widowed (*n* = 2226)**	${{\varvec{\chi}}}^{2}$	**P**
**Call time**					375.284	< *0.001*
Daytime	6186 (41.4%)	1586 (46.2%)	3441 (37%)	1159 (52.1%)		
First half of the night	5509 (36.8%)	1259 (36.6%)	3413 (36.7%)	837 (37.6%)		
Second half of the night	3260 (21.8%)	591 (17.2%)	2439 (26.2%)	230 (10.3%)		
^a^**Education level**					1464.997	< *0.001*
Low	1066 (8.2%)	428 (16.3%)	385 (4.7%)	253 (12.4%)		
Medium	2668 (20.6%)	574 (21.8%)	1189 (14.4%)	905 (44.4%)		
High	9210 (71.2%)	1628 (61.9%)	6702 (81%)	880 (43.2%)		
^a^**Occupation**					644.018	< *0.001*
Unemployed	7164 (51.2%)	992 (32.7%)	4739 (53.8%)	1433 (66.7%)		
Employed	6817 (48.8%)	2038 (67.3%)	4063 (46.2%)	716 (33.3%)		
^a^**Region**					1079.72	< *0.001*
Hangzhou	5147 (34.8%)	1280 (37.5%)	2480 (27.1%)	1387 (62.4%)		
Zhejiang province	3325 (22.5%)	895 (26.2%)	2117 (23.1%)	313 (14.1%)		
Other province	6331 (42.8%)	1241 (36.3%)	4569 (49.8%)	521 (23.5%)		

Some variables involved in the study may have missing data, and been marked “^a^”, resulting in varying numbers of callers for each variable, not encompassing the entire number of callers

### Comparison of call types from adult women callers of different marital statuses

Adult women callers of different marital statuses have distinct consulting patterns on hotline calls. Married callers mainly seek advice on marriage and family issues, child education, and physical problems. Single callers, on the other hand, primarily seek guidance on work-related matters, interpersonal relationships, and learning difficulties. Mental and psychological problems are common concerns among all three groups, with proportions of 44.7%, 57.6%, and 70.0% for married, single, and divorced/widowed callers, respectively (Table 5).

**Table 5:** Comparison of the types of calls from different marital statuses of adult women callers [PCS (%)]

Consultation issues	Total (*n* = 14949)	*Married① (*n* = 3435)	*Single② (*n* = 9290)	*Divorced or widowed③ (*n* = 2224)	Pairwise comparison *P* < 0.05
Physical illness	133 (0.9%)	57^a^ (1.7%)	61^b^ (0.7%)	15^b^ (0.7%)	① > ②③
Mental health issues	8444 (56.5%)	1536^a^ (44.7%)	5351^b^ (57.6%)	1557^c^ (70.0%)	① < ② < ③
Romantic relationship issues	1671 (11.2%)	20^a^ (0.6%)	1544^b^ (16.6%)	107^c^ (4.8%)	① < ③ < ②
Marital and family issues	1998 (13.4%)	1009^a^ (29.4%)	709^b^ (7.6%)	208^c^ (12.6%)	① > ③ > ②
Sexual issues	30 (0.2%)	9^a^ (0.3%)	17^a^ (0.2%)	4^a^ (0.2%)	
Parenting and child education issues	359 (2.4%)	279^a^ (8.1%)	8^b^ (0.1%)	72^c^ (3.2%)	① > ③ > ②
Work-related issues	567 (3.8%)	105^a^ (3.1%)	435^b^ (4.7%)	27^c^ (1.2%)	② > ① > ③
Interpersonal relationship issues	727 (4.9%)	136^a^ (4.0%)	521^b^ (5.6%)	70^a^ (3.1%)	② > ①③
Learning issues	246 (1.6%)	13^a^ (0.4%)	231^b^ (2.5%)	2^a^ (0.1%)	② > ①③
Other issues	774 (5.2%)	271^a^ (7.9%)	413^b^ (4.4%)	90^b^ (4.0%)	① > ②③

Individual variables have missing values, and been marked “*”,so the number of people will be less than the total. The results of pairwise comparison are shown in the table in alphabetic superscript format. If the superscript letter is the same, it means that there is no difference between the class column proportions; If the letters are different, there is a significant difference between the class column proportions

### Comparison of suicide risk and mental health status among adult women callers of different marital statuses

There are significant differences in the mental health and suicide risk among adult women callers of different marital statuses. Single women have higher rates of mild and moderate depression compared to married and divorced/widowed women. Single and divorced/widowed women also have higher rates of severe depression compared to married women. Additionally, single women have a significantly higher suicide risk compared to the other two groups. The mental health status of unmarried women is a cause for concern (Table 6).

**Table 6:** Comparison of suicide risk and psychological well-being among different marital statuses of adult women callers [PCS (%)]

Mental health factor	Total (*n* = 14955)	*Married① (*n* = 3436)	*Single② (*n* = 9293)	*Divorced or widowed③ (*n* = 2226)	Pairwise comparison *P* < 0.05
**Mood status**					
Normal	14321 (95.8%)	3310^a^ (96.3%)	8819^b^ (94.9%)	2192^c^ (98.5%)	② < ① < ③
Mild depression	180 (1.2%)	34^a,b^ (1.0%)	134^b^ (1.4%)	12^a^ (0.5%)	② > ③
Moderate depression	197 (1.3%)	27^a^ (0.8%)	159^b^ (1.7%)	11^a^ (0.5%)	② > ①③
Severe depression	257 (1.7%)	65^a^ (1.9%)	181^a^ (1.9%)	11^b^ (0.5%)	①② > ③
**Suicide risk**					
Have suicidal ideation	1878 (12.6%)	382^a^ (11.1%)	1403^b^ (15.1%)	93^c^ (4.2%)	② > ① > ③
Have suicide plan	88 (0.6%)	15^a,b^ (0.4%)	71^b^ (0.8%)	2^a^ (0.1%)	② > ③
*Suicide behavoir					
No	14450 (96.6%)	3368^a^ (98.0%)	8879^b^ (95.5%)	2203^c^ (99.0%)	③ > ① > ②
Used to be	460 (3.1%)	58^a^ (1.7%)	381^b^ (4.1%)	21^a^ (0.9%)	② > ①③
Preparing	12 (0.1%)	4^a^ (0.1%)	8^a^ (0.1%)	0^a^ (0.0%)	
Already	31 (0.2%)	5^a^ (0.1%)	25^a^ (0.3%)	1^a^ (0.0%)	
**High-risk call**	115 (0.8%)	21^a^ (0.6%)	92^a^ (1.0%)	2^b^ (0.1%)	①② > ③

Individual variables have missing values, and been marked “*”, so the number of people will be less than the total. The results of pairwise comparison are shown in the table in alphabetic superscript format. If the superscript letter is the same, it means that there is no difference between the class column proportions; If the letters are different, there is a significant difference between the class column proportions

### Comparison of mental health status among adult women callers at different call times

There are significant differences in the mental health status of adult women callers depending on the time they make their calls. Nighttime calls have higher proportions of mild and moderate depression, suicidal plans, and high-risk individuals, especially in the second half of night. Interestingly, from the data, it can be seen that the proportion of severe depression callers was significantly higher for daytime calls than nighttime calls. Callers with suicidal ideation and a history of suicide attempts also tended to call during the day (Table 7).

**Table 7:** Comparison of psychological well-being among adult women callers based on call time [PCS (%)]

Mental health factor	Total (*n* = 15580)	Daytime① (*n* = 6328)	First half of night② (*n* = 5785)	Second half of night③ (*n* = 3467)	Pairwise comparison *P* < 0.05
**Mood status**					
Normal	14928 (95.8%)	6102^a^ (96.4%)	5532^b^ (95.6%)	3294^b^ (95.0%)	① > ②③
Mild depression	185 (1.2%)	22^a^ (0.3%)	99^b^ (1.7%)	64^b^ (1.8%)	① < ②③
Moderate depression	202 (1.3%)	67^a^ (1.1%)	78^a,b^ (1.63%)	57^b^ (1.6%)	① < ③
Severe depression	265 (1.7%)	137^a^ (2.2%)	76^b^ (1.3%)	52^b^ (1.5%)	① > ②③
**Suicide risk**					
Have suicidal ideation	1937 (12.4%)	1041^a^ (16.5%)	514^b^ (8.9%)	382^c^ (11.0%)	① > ③ > ②
Have suicide plan	91 (0.6%)	25^a^ (0.4%)	29^a^ (0.5%)	37^b^ (1.1%)	①② < ③
*Suicide behaviour					
No	15,057 (96.7%)	6044^a^ (95.5%)	5669^b^ (98.0%)	3344^c^ (96.5%)	① < ③ < ②
Used to be	474 (3.0%)	270^a^ (4.3%)	99^b^ (1.7%)	105^c^ (3.0%)	① > ③ > ②
Preparing	12 (0.1%)	3^a^ (0.0%)	5^a^ (0.1%)	4^a^ (0.1%)	
Already	35 (0.2%)	11^a^ (0.2%)	11^a^ (0.2%)	13^a^ (0.4%)	
**High-risk call**	121 (0.8%)	31^a^ (0.5%)	49^b^ (0.8%)	41^b^ (1.2%)	① < ②③

Individual variables have missing values, and been marked “*”, so the number of people will be less than the total. The results of pairwise comparison are shown in the table in alphabetic superscript format. If the superscript letter is the same, it means that there is no difference between the class column proportions; If the letters are different, there is a significant difference between the class column proportions

### Logistic regression analysis on the depressive status of adult female callers

The scores obtained from the assessment of emotional state were transformed into a binary variable, which served as the dependent variable (depression level). Sociodemographic variables such as age, marital status, education level, etc., were included as independent variables in the multivariate logistic regression. The variable assignments can be found in Table 8.

**Table 8:** Assignment of variables

Variable	Encode	Assignment
Age	X1	≤ 30 = 1; > 30 = 2
Marital Status	X2	married = 1; Single = 2; Divorced / widowed = 3
Education level	X3	low = 1; medium = 2; high = 3
Call time	X4	daytime = 1; first half of the night = 2; second half of the night = 3
Occupation	X6	unemployed = 0; employed = 1
Mood status	Y1	lighter = 0; heavier = 1
High-risk call	Y2	no = 0; yes = 1

The results revealed the following associations: an increase in age category decreased the risk of depression significantly (OR = 0.263, 95%CI:0.195–0.355,* P* < 0.001). Compared to women with low education levels, those with moderate education levels had a lower risk of higher depression levels (OR = 0.633, 95%CI:0.416–0.964, *P* = 0.033), as did women with high education levels (OR = 0.631, 95%CI:0.439–0.906, *P* = 0.013). Women who were employed had a lower risk of higher depression levels compared to those unemployed (OR = 0.632, 95%CI:0.423–0.944, *P* = 0.025). Interestingly, callers who made contact during the night had a lower risk of higher depression levels compared to daytime callers. The odds ratios were 0.770 (95%CI:0.613–0.968, *P* = 0.025) for the first half of the night and 0.692 (95%CI:0.529–0.906, *P* = 0.007) for the second half of the night (Please refer to Table 9).

**Table 9:** Logistic regression analysis on the depressive status of adult female callers [0R(95%CI)]

**Variable**	**Group**						OR 95%CI	
		B	B-SD	Wald	P	OR	Lower limit	Upper limit
Age	≤ 30^a^							
	> 30	-1.334	0.152	77.01	< *0.001*	0.263	0.195	0.355
Marital Status	married^a^							
	Single	-0.245	0.152	2.602	*0.107*	0.783	0.581	1.054
	Divorced / widowed	-0.67	0.263	6.507	*0.11*	0.512	0.306	0.856
Education level	low^a^							
	medium	-0.457	0.215	4.537	*0.033*	0.633	0.416	0.964
	high	-0.461	0.185	6.202	*0.013*	0.631	0.439	0.906
Call time	daytime^a^							
	first half of the night	-0.261	0.117	5.029	*0.025*	0.77	0.613	0.968
	second half of the night	-0.368	0.137	7.177	*0.007*	0.692	0.529	0.906
Occupation	unemployed^a^							
	employed	-0.459	0.205	5.022	*0.025*	0.632	0.423	0.944

“^a^” stands for “Control group”

#### Factors influencing high-risk calls from adult females

The dependent variable was whether the call was considered high-risk, with values of 0 for “no” and 1 for “yes”. Several variables were examined to understand their impact on high-risk calls made by adult women. Factors such as emotional state, time of call, education level, employment status, and marital status were found to be significant (*P* < 0.05).

Higher levels of depression were associated with increased odds of a high-risk call, those with mild depression had a 20.953 times higher risk (*P* < 0.001), those with moderate depression had a 108.292 times higher risk (*P* < 0.001), and those with severe depression had a 1107.253 times higher risk (*P* < 0.001).

Nighttime calls, especially in the second half of the night, had significantly higher odds of being high-risk compared to daytime calls. the odds of a high-risk call is 2.485 times higher for callers in the first half of the night (*P* < 0.001) and 5.326 times higher for callers in the second half of the night (*P* < 0.001). Higher education levels were linked to a lower risk of high-risk calls (OR = 0.328, 95%CI:0.147–0.733, *P* = 0.007). Additionally, single callers had a slightly higher risk compared to married callers (OR = 2.043, 95%CI:1.001–4.221, *P* = 0.05) as shown in Table 10.

**Table 10:** Factors influencing high-risk calls from adult females [0R(95%CI)]

**Variable**	**Group**						OR 95%CI	
		B	B-SD	Wald	P	OR	Lower limit	Upper limit
Mood status	Normal^a^			290.523	< *0.001*			
	mild depression	3.089	0.7	19.462	< *0.001*	21.953	5.565	86.593
	Moderate depression	4.694	0.489	92.326	< *0.001*	109.292	41.952	284.719
	severe depression	7.011	0.425	272.213	< *0.001*	1108.254	481.898	2548.73
Call time	Daytime^a^			24.678	< *0.001*			
	first half of the night	1.248	0.315	15.729	< *0.001*	3.485	1.88	6.459
	second half of the night	1.673	0.36	21.646	< *0.001*	5.326	2.633	10.775
Education level	Low^a^			8.843	*0.012*			
	medium	-0.536	0.476	1.267	*0.26*	0.585	0.23	1.488
	high	-1.115	0.411	7.372	*0.007*	0.328	0.147	0.733
Occupation	Unemployed^a^							
	employed	-0.624	0.263	5.622	*0.018*	0.536	0.32	0.897
Marital Status	Married^a^			6.797	*0.033*			
	Single	0.714	0.37	3.725	*0.05*	2.043	1.001	4.221
	Divorced / widowed	-0.874	0.866	1.019	*0.313*	0.417	0.076	2.277

“^a^” stands for “Control group”

## Discussion

### Basic characteristics of adult female callers

The majority of female callers are relatively young, with a higher level of education, and unmarried. More than half of the callers are below 30 years old, with an unmarried ratio of 89.4% and 83% having a higher education level, aligning with findings from international studies [26]. The data indicates that young unmarried women with higher education levels have a higher prevalence of depressive mood, suicide risk, and high-risk callers compared to older women. This suggests that young women experience more severe psychological distress and seek social support. Their education level drives them to prioritize mental health and actively seek help through various channels. This emphasizes the benefits of hotlines, which provide remote psychological services, identify potential callers in need, and offer timely referrals for diagnosis and treatment.

### Types of consultation issues for adult female callers

This study shows that mental and psychological problems are the main consultation topics for adult female callers, which is consistent with other research findings [27]. Females tend to experience more negative emotions, which puts them at a higher risk of developing mental and psychological problems [28]. Research indicates that females are more prone to emotional fluctuations and are more likely to express their emotions in extreme ways compared to males [29]. Mental and psychological disorders have the highest burden of disease in China, accounting for about 20% and increasing over time [30]. The intensifying social competition adds to the psychological burden, especially for females. This highlights the importance of helplines providing professional support and enhancing counselor training in mental health, specifically in depression. Identifying callers with possible depression or other mental disorders and offering timely professional advice and referrals is a critical objective of helpline services.

Additionally, Young women seek advice on romantic relationships more often than those aged 30 and above. Among women aged 30 and above, especially married women, seeking advice on marriage, family, and child education is more common. These findings reflect societal realities. To effectively help adult females, helpline counseling services should focus on improving their ability to address these prevalent issues, in addition to mental health problems. This support will assist callers in resolving internal conflicts and negative emotions related to these problems.

### Psychological well-being of adult female callers

#### Impact of marital status

This study found that single women have poorer mental health, higher depression levels, and a greater risk of suicidal thoughts or previous attempts. These outcomes may be attributed to the impact of the COVID-19 pandemic. Industries predominantly employing women, such as airlines, hotels, childcare, restaurants, and retail, were significantly affected by the economic downturn, leading to job losses and financial hardships [31]. Additionally, home isolation measures resulted in heightened loneliness for unmarried women. The combined effects of fear, anxiety, loneliness, and economic and social pressures have led to a mental health crisis among this group [32].

**Fig. 1:**
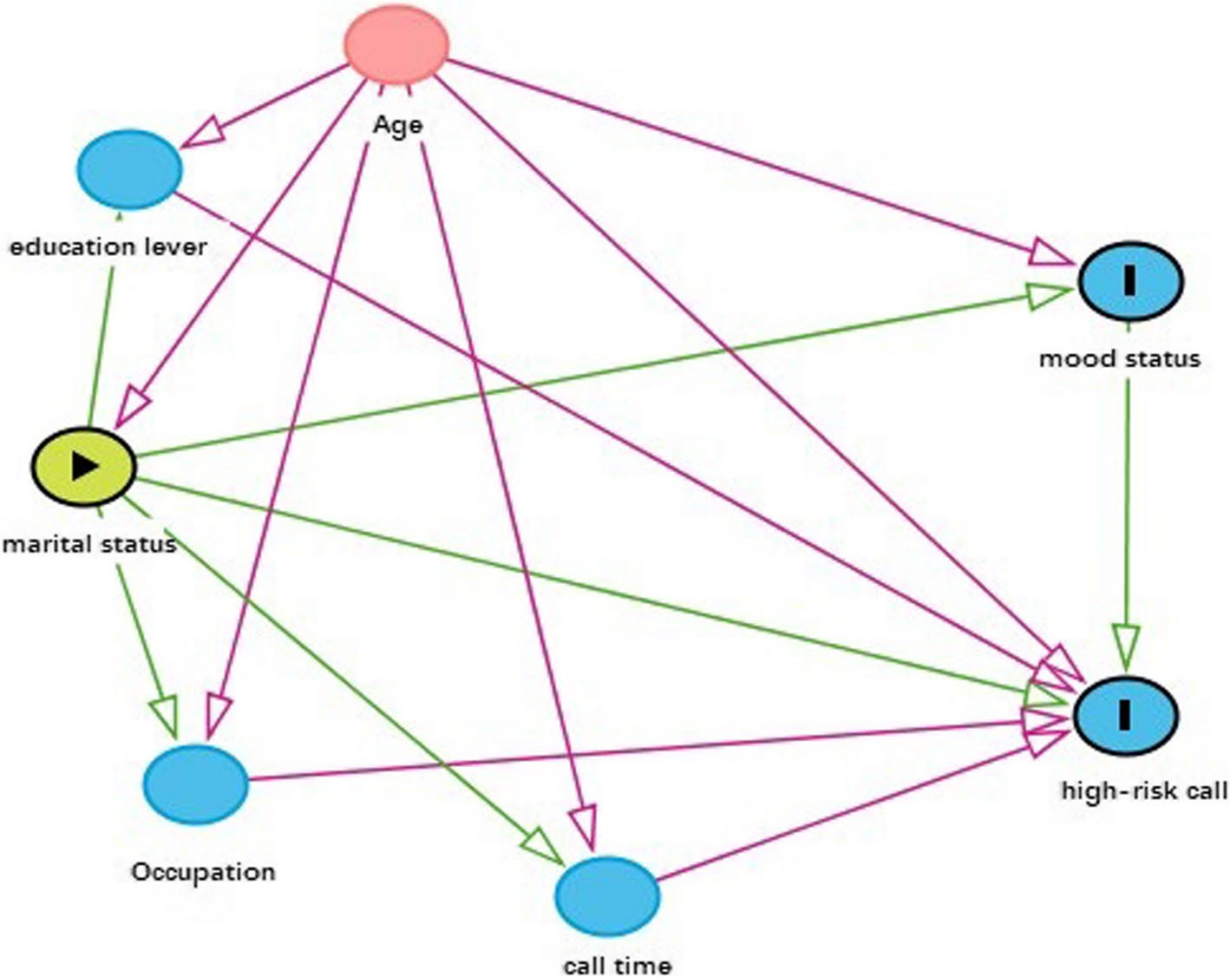
The directed acyclic graph (DAG)

It's important to note that there was a statistical difference in marital status when conducting a univariate analysis of depression levels in adult females (Table 11). However, this “association” disappeared in the multivariate analysis. Based on our experience, we have found a strong correlation between the “age” factor and the “marital status” factor through two-factor analysis and directed acyclic graph (Fig. 1). There is evidence of collinearity between these two factors. In multiple factor analysis, when adjusting for the influence of the “age” factor, the “association” between the “marital status” factor and the dependent variable disappears. Previous research indicates that the influence of marriage on emotional state is primarily reflected in competence and quality [33]. In modern society, influenced by various political, economic, and cultural factors, the stability of marriage has been greatly impacted. Compared to traditional society, modern marriage resembles or is a special “profession” that requires individuals entering this special “profession” to possess basic psychological qualifications or abilities. Adults can autonomously choose their own lifestyle [34]. Therefore, studying the influence of marital status on emotional state solely based on “married” or “unmarried” is insufficient. In the future, more targeted research focusing on individuals themselves is needed to provide more guiding and constructive interventions for callers.

**Table 11:** The effect of marital status on the degree of depression

	B	B-SD	Wald	F	P	Exp(B)	EXP(B) 95% CI	
							Lower	Upper
Marital status	-.204	.078	6.749	1	.009	.816	.699	.951
Constant	-3.080	.153	406.926	1	.000	.046

#### The influence of call time

Individuals with severe depression and suicidal thoughts or attempts are more inclined to make daytime calls rather than nighttime calls. This pattern may be connected to women's emotional tendencies, as research suggests they have a higher inclination for expressing and sharing emotions compared to men. Among them, middle-aged women have the highest score in emotional expression [35]. Considering the available data, With the proportion of daytime calls reaching 40.6%, it is understandable why suicidal ideation, which stems from negative emotional expression, is more prevalent during the day based on the available data. Various factors, including biological rhythms, daily events, and social interactions, can influence a person’s emotional state. Poor sleep quality or limited emotional regulation upon waking up may contribute to lower emotional well-being in the morning. Daytime, being an active period with work, study, and social interactions, can trigger emotional distress and negative feelings, particularly for women, prompting them to seek psychological assistance and support.

For high-risk callers, the time of the call is significantly correlated with their mental health status. From the data results in Table 10, we can see that compared to daytime callers, those who call during the late night have a 5.326 times higher risk of high-risk behavior. Previous research has indicated that individuals are more likely to feel a lack of social support, vulnerability, hopelessness, engage in catastrophic thinking, and have low impulse control during the early morning hours, increasing the risk of suicide [36]. Additionally, the early morning is a high-risk period for suicides. Individuals who make hotline calls during this time may have sleep disturbances or poor sleep due to depressive emotions, which may contribute to poor mental health among female callers during this time [37]. Therefore, it is necessary to strengthen the assessment of negative emotions and suicide risk among female callers during the early morning to prevent suicidal behavior.

#### Influence of education level

This study shows that 71.3% of female callers have a higher education level, with more than half of them being unmarried and under the age of 30. Research has demonstrated that there are statistically significant differences in emotional effects among women with different education levels [38]. Higher educational attainment is associated with more stable emotions and a greater tendency to express emotions. Compared to a lower education level, a higher education level can reduce the risk of high-risk calls. This result is consistent with other research findings [39]. Higher education levels have several positive effects on individuals. Firstly, they enhance cognitive abilities, enabling better problem-solving, reducing depressive emotions, and promoting personal health, including exercise habits and lower rates of chronic diseases. Secondly, higher education correlates with higher income and social status, providing resources for improved personal health, increased life satisfaction, and reduced psychological and emotional problems, ultimately lowering depression levels. Additionally, previous research has shown that married individuals with higher education often have partners with similar educational backgrounds, which means that people with higher education levels are more likely to understand and empathize with each other [40]. The impact of education on depression is mainly achieved through enhancing cognitive abilities and physical health levels [41, 42]. Therefore, our hotline work needs to strengthen health knowledge promotion and education for the general public, enhance public health literacy, foster good exercise habits, and align with school education to jointly promote the improvement of social mental health levels.

#### Impact of employment status

Unemployment is a risk factor for high-risk callers, as found in this study. Unemployed women can be classified into four types based on social role division and marital status: actively chosen married, passively accepted married, actively chosen unmarried, and passively accepted unmarried. Actively chosen married women’s psychological issues stem from family dynamics and they experience mild depressive emotions. Hotline counselors primarily listen and provide emotional guidance. Actively chosen unmarried women, often students, face academic and interpersonal challenges. Hotline counseling should address these issues using techniques from short-term interpersonal psychotherapy. Counselors with career planning knowledge can help address negative emotions related to future development among this group.

Worth focusing on the passively accepted married and unmarried women, as they experience greater psychological pressure and a higher risk of suicide. Being passively accepted implies that they may have suffered from the blow of unemployment. McKinsey report data shows that during the COVID-19 pandemic, women faced a 1.8 times higher risk of unemployment compared to men [43]. While women represent 39% of global employment, they accounted for 54% of total job losses, and the post-pandemic return-to-work rate for women is lower than that of men. Furthermore, research indicates that young women facing economic difficulties due to job loss or income loss are more prone to negative emotions such as anxiety compared to their male counterparts [44].

For women with families and children, passive acceptance may also result from the conflict between work and family, leading them to sacrifice their jobs for the sake of the family. For example, 2020 McKinsey report on working women showed that one-third of mothers considered reducing career expectations or leaving their jobs during the pandemic [45]. Their vulnerable economic status and strong family dependency exacerbate their psychological burden, resulting in an increasing prevalence of depression and a significant increase in suicide rates among this group. Therefore, conducting psychological interventions for unemployed women through hotlines is of significant importance in preventing and reducing suicide rates among this population. Currently, in China, hotline interventions are limited to assessing and relieving immediate emotions [46], but it would be beneficial to expand the hotline services to provide tailored interventions for this specific group.

## Conclusion

This study concludes that unemployed women with lower education levels who make late-night calls are at higher risk for poor mental health and suicide. They need special attention, targeted services, and interventions to ensure appropriate mental health support. However, currently, there is a clear gender disparity in research on health, with women being in a disadvantaged position [47]. The main challenges faced by women are low disease awareness and difficulties in accessing medical resources. To address women's mental health issues, the government should adjust the allocation of medical resources accordingly, from both economic and public health standpoints.

Overall, this study emphasizes the importance of hotline counseling for addressing psychological issues among adult female callers in China. The nationwide distribution of callers indicates the sample’s representativeness and the significant impact of these services at a national level. Hotline counseling offers distinct advantages in overcoming barriers associated with traditional services, making it a valuable tool for providing psychological support, assessments, and referrals, ultimately enhancing the mental health outcomes of female callers in need.

The study has limitations: 1) The hotline’s structured counseling limits the collection of detailed individual information on factors like marital satisfaction, physical health, and family economic status. 2) The study focused on COVID—19 during a pandemic, may limit the results of wider applicability. 3) The study focused only on depressive symptoms and suicidal behaviors, lacking a comprehensive assessment of mood. 4) Being a retrospective study, it cannot establish cause and effect. Future interventional studies are needed to explore risk factors and develop targeted interventions such as dialectical behavior therapy and cognitive behavior therapy. These interventions can be implemented through the hotline to reduce depression and suicide risk among Chinese women.

### Supplementary Information

**Additional file 1. **

## Data Availability

The datasets generated during the current study are not publicly available due Involving some personal information about the caller, but are available from the corresponding author on reasonable request.
