# Supplementary material for: Adult female callers’ characteristics and mental health status: a retrospective study based on the psychological assistance hotline in Hangzhou
Source: BMC Public Health. 2023 Nov 20;23:2295. doi: 10.1186/s12889-023-17085-6 (PMC10662539; doi:10.1186/s12889-023-17085-6)
Supplement: Supplementary file 1 — Additional file 1. [file 12889_2023_17085_MOESM1_ESM.docx]

Additional file 1

**Depression and Suicide Risk Assessment Questionnaire**

Date of call: Time: Number: Caller ID: Counselor:

| 1 Have you felt down or depressed recently? (sad face, sighing, tears alone) Yes (duration) No |
| --- |
| 2 Have you recently lost interest in things you normally enjoy? Yes (duration ) No |
| 3 Have you recently felt worthless, a useless person or a failure? Yes No |
| 4 Do you blame yourself or have regrets? Yes No |
| 5 Has your weight changed recently? Yes (increase decrease) No  Any recent changes in your appetite Yes (increase decrease) No |
| 6 Has your sleep changed recently? Yes (increase decrease) No |
| 7 Have you had any physical complaints recently? (Dizziness, fatigue, listlessness or lack of energy) Yes No |
| 8 Have you recently had trouble concentrating, thinking flexibly, or being stupid? Yes No |
| 9 Did others see that you were different than usual (expression, speech, behavior, reaction)? Yes No |
| 10 Has the above problem affected your work, study, life, socialization, etc.? Yes No |
| 11 Do you feel distressed when these problems arise? Yes No |
| 12 Many people have thought about death when they are in the above situations; have you ever thought about harming yourself? Yes No |
| 13 Do you have a specific plan for self-injury or suicide? No Yes (describe: )  What is the intended time frame for this program? No Yes (describe: )  Do you really want to die? Yes No |
| 14 Have you ever committed suicide or intentionally harmed yourself before? No Yes (describe: )  How many suicidal behaviors were there? Times.  When did this last happen? Year Month Day  Did you really want to die at that time? Yes No |
| 15 Have any of your relatives, friends, co-workers, or acquaintances ever self-injured or committed suicide? Yes No |
| 16 If "0" means no hope and "100" means the most hope, how hopeful are you about your future life? |
| 17 In the past month, have you had any serious physical illnesses, major life events? Yes No |
| 18 In the last month, have you consumed excessive alcohol, abused sleeping pills, narcotics or stimulants? Yes No |
| 19 What are the main causes of your suicidal thoughts? |
| 20 What problem do you hope to solve or achieve through suicide? |

Judging Criteria:

No depression: 1, 2 rated no, no suicidal ideation.

Mild depression: one of 1 or 2 present, 4 of 1-9 present, 10 social functioning impaired or 11 caller distressed.

Moderate depression: one of 1 or 2 present, 5-7 of 1-9 present, 10 social functioning impaired or 11 caller distressed.

Major depression: 1 or 2 with one present for 1 week duration, 7 or more of 1 to 9, 10 social functioning impaired or 11 caller distressed.

**High-risk callers: ① with suicidal ideation and a specific suicide plan; ② with suicidal ideation without a plan but with a risk factor score ≥ 5.**

Risk factors: 1 point for each of 14 and 15 responses, and 1 point for ≤50% hopefulness for 16.

17, 18 One point for the presence of a physical illness, life event, or substance abuse.

1 point for moderate depression and 2 points for severe depression.

**Assessment: Mild Moderate Severe High Risk**
